# Meetings of Societies

**Published:** 1905-12

**Authors:** 


### MEETINGS OF SOCIETIES.

### Bristol Medico-Chirurgical Society.

*Annual Meeting, October 11th, 1905.*

After a vote of thanks to the retiring president, Dr. Reginald Eager, had been unanimously passed, Mr. John Dacre took the chair and gave an introductory address (see p. 289). Later a cordial vote of thanks was given to Mr. Dacre for his able and eloquent address.

The honorary secretary, Mr. H. F. Mole, read his annual report. The balance in hand was £162 16s. 2d. on the ordinary account, and £11 14s. 10d. on the *Journal* account. A large grant had been made to the library.

The editorial secretary, Mr. James Taylor, read his annual report, which was followed by that of Mr. C. K. Rudge, the honorary librarian of the Medical Library. The latter report showed that the Bristol Medical Library now contained 21,274 volumes, and was in receipt of 226 current periodicals. These reports were adopted.

The following officers were chosen for the ensuing year: President-Elect, Mr. James Taylor; Members of Committee, Dr. J. Michell Clarke, Dr. B. M. H. Rogers, Dr. H. Waldo, Dr. P. Watson Williams, Mr. C. K. Rudge, and Dr. James Swain; and Members of the Library Committee, Mr. C. K. Rudge, Mr. Munro Smith, and Mr. T. Carwardine.

*November 8th, 1905.*

Mr. JOHN DACRE, President, in the Chair.

Dr. J. O. SYMES showed a patient who had been **injured by lightning.**

Dr. CHARLES showed a patient with paresis of the arms, the result of **syphilitic disease in the cervical region of the spinal cord and membranes.**

Dr. KENNETH WILLS read a paper on Soap in Skin Practice. He described certain tests he had applied to thirty varieties of soap advocated for skin diseases. Soaps which take the longest time to dry are the least irritating. Those which most quickly form bubbles on an inverted shaving brush are the most irritating. The quality and quantity of the substances left on the skin after rinsing away the soap determined the amount of irritation produced. Soap should leave a bland oil on the surface without free alkali. He showed specimens of soap of good quality which he had had made by a firm of soap manufacturers and which he now recommended when asked for advice in the matter.—Dr. J. O. SYMES alluded to some investigations he had carried out to ascertain how far antiseptics retained their properties when incorporated with soap. Antiseptic properties he had found were lost by carbolic acid, and that of the antiseptics tested biniodide of mercury best retained its bactericidal properties.—Mr. MUNRO SMITH did not accept the tests described by Dr. Wills as conclusive. He thought that the rancid odour often detected near soap works indicated that very bad fat was used in the manufacture.—Dr. WILLS said that he thought no acid incorporated with soap would remain free, but would unite with the alkali in solution. Soap did not bubble so quickly if superfatted.

Dr. W. H. C. NEWNHAM read notes of a case of **ectopic pregnancy**, and showed the specimen obtained from the patient. The patient was 38, had been married ten years, and had not been pregnant before. At the end of July she had been suddenly seized with abdominal pain and became seriously collapsed, and in this state was seen by Dr. Langridge. She was not operated upon until October 7th. There was then a swelling in the middle line of the abdomen. A fœtus of four or five months of age was found on laparotomy, in connection with the enlarged right Fallopian tube. The patient made an excellent recovery.—Dr. LANGRIDGE pointed out that appendicitis had to be considered as a possible cause of the illness when he had first seen the patient, but he had concluded that ectopic gestation was the cause of the symptoms. In considering the treatment of such cases regard must be paid to the difficulties and dangers attending operation in country districts far removed from hospitals and nursing homes.

Dr. WALTER SWAYNE read a paper on **ectopic gestation** and showed specimens. Three cases were described, in all of which recovery had followed operation. In one of these the tube removed was distended but not ruptured. The speaker remarked that it is when the hemorrhage is small that there is doubt as to the necessity of immediate operation, and the accessibility of the patient is then to be taken into consideration.—The PRESIDENT alluded to a case of ectopic gestation he had met with in his practice. Pain in the groin following a fall had been first complained of. A few days later very severe pain was experienced, and a large swelling formed behind the uterus. Tubal pregnancy was diagnosed and the patient was removed to a surgical home for operation. During the night the patient aborted, but next morning the operation was proceeded with by Mr. Mole. The patient recovered.—Mr. MOLE thought this case was of exceptional interest because there had been both intra- and extra-uterine gestation at the same time.—Mr. PAUL BUSH alluded to a case in the Bristol Royal Infirmary in which a woman was admitted with a history suggesting miscarriage. The following day the patient suddenly became worse, and though operation was then performed within a few minutes the patient could not be saved. Such a case showed the danger of waiting even when everything was ready for quickly operating if the patient suddenly changed for the worse.—Dr. GREEN mentioned a case in which the patient had recovered without operation. He thought, nevertheless, that treatment by operation was the only safe one.—Dr. NEWNHAM concluded that for 99 out of 100 cases the only proper treatment was by operation. He had only himself seen one fatal case.

Dr. FORTESCUE-BRICKDALE read a short paper on **anti-tuberculous vaccines and sera**, the former being attempts to produce an active, and the latter a passive immunisation against the disease. Vaccines might be classed in three groups, the first containing extraction products of the bacilli (bacterio-proteins), and resembling Koch's old tuberculin; the second containing disintegration products (bacterio-plasmins), of which Koch's bacterial emulsion was a type. The third class contained a few miscellaneous products not falling under either of the two first heads, such as Béraneck's tuberculin. The experimental and clinical facts concerning tuberculins were then dealt with, and their essential limitations indicated. The antituberculous sera were next described, of which the most important were Maragliano's and Marmorek's. The former was now somewhat discredited, but the latter appeared capable of doing good in certain cases, though too weak to be considered an actual specific. Finally, Behring's new protective substance was described, as far as is at present possible.

Dr. P. WATSON WILLIAMS alluded to the difficulties of tracing the connection between remedies and results in the treatment of tuberculous diseases. He had tried various sera. The patients usually reported themselves as feeling better, but no sera had gained his confidence. In lupus he had had very good results in a certain number of cases, *e.g.* lupus affecting the larynx, nose and cornea. But the effects of sanatorium treatment were far better than those of any sera.

J. Lacy Firth.
H. F. Mole, *Hon. Sec.*

